# Malpractice—Two Cases

**Published:** 1879-07

**Authors:** 

**Affiliations:** Oxford, Ga.


### MALPRACTICE—TWO CASES.

---

*Editor Medical and Surgical Journal:*

Mrs. —— was delivered of one child by a colored midwife, on the 12th April, 1878, a twin presenting preternaturally; I, nevertheless, was not called on until the next day. Found pains active, shoulder presenting, funis extended and pulseless. Pelvis large and child relatively small. By aiding the tendency to "spontaneous evolution" breech delivery of a dead child was easily and speedily effected. The athletic mother soon rebounded into full health without an adverse symptom. After a few months, healthy menstruation began and continued until the succeeding December, when it ceased. In due time the usual symptoms of pregnancy came on; she being as perfect a picture of heath, and as free from signs of occult disease as any woman in her condition could possibly be. But unfortunately, an epidemic, or rather endemic prevailed in the public mind of a certain class. (Why, how, or by whom started, need not be here stated.) Womb "have got very much out of fix," and need doctoring. The female genetalia had lost their sanctity. The shrinking modesty and delicate reserve of blushing womanhood must yield to the ignorant neophite or rash and clamorous demands of the boastful pretender, or death must ensue. For now it has become an exploded dogma, that the educated touch of the obstretrician, supercedes, for the most part, the use of the eyes in such cases. Formerly, with certain ones, all obscure diseases were easily and safely traced to that grand depository of all human ills and errors, the liver. But now, the womb has become the receptacle of maladies known and unknown, and suffer in its way, as the liver does, when made a hydrargyric target. The dusky patient, fully infected with the prevailing mania, was charged to disclose, instead of conjugal embraces, the turbulent upheavals of her spasmodic stomach. A son of Æsculapias suited to the emergency must be had, and unluckily, was soon found; and then *he found* that the uterus had been behaving badly for more than six months. It had something in it, not a special deposit by the salacious husband, but a malady, whose name was "of learned length and thundering sound." The doctor could say *veni, vidi visi.* The husband did say the doctor used something like a "glove-stretcher" to open the mouth of the womb, and with another instrument inserted a batch of something and left it there. The offended viscus resisting the wrong, thrust into the world, to the amazement of the mother, and the disgrace of the doctor, a six and a half month's child, who, after a few feeble gasps, died of the ignorant interference, that hastened its advent. I am credibly informed a similar case occurred a few month's ago, in the same vicinity, but in other hands. Now, is this infanticide, or is it not? Whatever it may be, it certaily is not justifiable homicide.

Of course, no fling is intended at the illustrious men who adorn their calling, and bestow such benefactions on suffering humanity, in this and other departments of gynæcology.

A Couvert, M.D.

*Oxford, Ga, July,* 1879.

