# Errata

**Published:** 1879-10

**Authors:** 


*Mr. Editor:*

The article "Malpractice" was sent so late and inserted so hastily in the JOURNAL of July that several errors crept in, some of which may bring me into disrepute among my professional brethren. Will you therefore please do me the favor to publish the following corrections, and greatly oblige

Very respectfully yours, A COUVERT M.D.

ERRATA.

On page 245, 4th line from top, for "extended" read extruded; 16th line from top, for "womb have" read wombs had; 6th line from bottom, for "suffer" read suffers; 3d line from bottom, for "disclose" read disease; 4th line from bottom, for "erase" read was. Page 246, 5th line from top, for "visi" read vici; 9th line from top, for "resisting" read resenting; 15th line from top, for "certaily" read certainly.

